# Synthesis and Application of Scaffolds of Chitosan-Graphene Oxide by the Freeze-Drying Method for Tissue Regeneration

**DOI:** 10.3390/molecules23102651

**Published:** 2018-10-16

**Authors:** Cesar Valencia, Carlos H. Valencia, Fabio Zuluaga, Mayra E. Valencia, José H. Mina, Carlos David Grande-Tovar

**Affiliations:** 1 Laboratorio SIMERQO polímeros, Departamento de Química, Universidad del Valle, Calle 13 No 100-00, 76001 Cali, Colombia; cesar.valencia@correounivalle.edu.co (C.V.); hector.zuluaga@correounivalle.edu.co (F.Z.); 2 Escuela de Odontología, Grupo biomateriales dentales, Universidad del Valle, Calle 13 No 100-00, 76001 Cali, Colombia; carlos.humberto.valencia@correounivalle.edu.co; 3 Grupo de Materiales Compuestos, Escuela de Ingeniería de Materiales, Universidad del Valle, Calle 13 No 100-00, 76001 Cali, Colombia; valencia.mayra@correounivalle.edu.co (M.E.V.); jose.mina@correounivalle.edu.co (J.H.M.); 4 Grupo de Investigación de Fotoquímica y Fotobiología, Universidad del Atlántico, Carrera 30 No 8-49, 081008 Puerto Colombia, Colombia

**Keywords:** chitosan, graphene oxide, freeze-drying method, scaffolds

## Abstract

Several biomaterials, including natural polymers, are used to increase cellular interactions as an effective way to treat bone injuries. Chitosan (CS) is one of the most studied biocompatible natural polymers. Graphene oxide (GO) is a carbon-based nanomaterial capable of imparting desired properties to the scaffolds. In the present study, CS and GO were used for scaffold preparation. CS was extracted from the mycelium of the fungus *Aspergillus niger*. On the other hand, GO was synthesized using an improved Hummers-Offemann method and was characterized by Fourier transform infrared spectroscopy (FTIR), Raman spectroscopy, atomic force microscopy (AFM), X-ray diffraction (XRD), and dynamic light scattering (DLS). Subsequently, three formulations (GO 0%, 0.5%, and 1%) were used to prepare the scaffolds by the freeze-drying technique. The scaffolds were characterized by FTIR, thermogravimetric analysis (TGA), and scanning electron microscopy (SEM), to determine their thermal stability and pore size, demonstrating that their stability increased with the increase of GO amount. Finally, the scaffolds were implanted, recollected 30 days later, and studied with an optical microscope, which evidenced the recovery of the tissue architecture and excellent biocompatibility. Hence, these results strongly suggested the inherent nature of chitosan/graphene oxide (CS/GO) scaffolds for their application in bone tissue regeneration.

## 1. Introduction

Nowadays, the “gold standard” for repairing of bone injuries included the use of either an autograft or an allograft; however, these have presented some drawbacks associated with limited supply of bone from the host, donor site pain, potential for donor site infection for an autograft, high cost, and the risk of viral and bacterial transmission for an allograft [1,2,3]. Bone tissue engineering as an effective way to treat bone injuries, has attracted widespread interest from researchers in recent years [4]. Several biomaterials, including natural and synthetic polymers, are used to study cellular interaction, proliferation, and differentiation [5]. Bone tissue recovery is parallel with biochemical processes that occur at the molecular level and are reflected in cell development. Therefore, it is appropriate to encourage the growth of cells by creating new materials with scaffolds characteristics [6].

A scaffold is a support that allows cellular interactions, which contribute to the formation and repair of functional tissues [7]. Therefore, scaffolds act as supports to facilitate the migration, adhesion, and transport of bioactive cells or molecules in charge of regenerative processes [7]. The scaffolds must, therefore, be three-dimensional and porous structures that serve as a temporary cellular matrix to promote vascular and cellular growth, while the expected tissue regeneration takes place [8].

One of the most promising polymeric materials seems to be chitosan (CS), a deacetylated chitin-derived polysaccharide, reported to be a safe, hemostatic, biocompatible, osteoconductive promoter of the mineralized bone matrix [9], and provider of minimum inflammatory responses after implantation [9]. The similarity between chitosan and glycosaminoglycans structure, a component of the extracellular matrix, facilitates interaction with cells, promoting colonization and encouraging cell differentiation and maturation, [6]. However, the brittle nature and poor dimensional stability of the CS scaffold limits its application.

On the other hand, graphene oxide (GO) is a resistant two-dimensional structure molecule, with the thickness of a carbon atom, but with high strength due to the σ-type bonds and the resonance characteristics in the π-type bonds of the carbons with hybridization sp^2^. GO also presents high stability and properties of a low annular tension aromatic polycycle [10].

The GO exhibits a sp^3^ hybridization that is related to the oxygenate groups in the vicinity and the central part of the sheet [11]. The oxygenated groups obtained in the GO by oxidation of graphene consist of hydroxyl (OH), epoxy (COC), and carboxyl (COOH), which gives the GO the possibility of hydrogen bonding to other molecules, such as CS, which in turn presents amine (NH_2_) and hydroxyl (OH) groups [11].

Functional moieties such as hydroxyl and carboxyl groups, have been reported to form chemical bonds with CS in scaffold matrices, thereby improving the biological stability and interfacial strength of scaffolds [4,12,13]. Therefore, the introduction of GO into the CS compound should reinforce the mechanical and thermal properties of the scaffold [14].

In the literature, there are only a few reports on the formation of CS/GO composites with a well-organized structure for bone tissue engineering applications [15]. However, to the best of our knowledge, there are no reports including the synthesis and characterization of GO with an improved method, isolation of CS from the mycelia of *Aspergillus niger* and the implantation of the scaffolds in rats’ skin, producing normal tissue recovery that evidence biocompatibility of the material.

## 2. Results and Discussion

### 2.1. CS Extraction from the Mycelium of A. niger

The basic hydrolysis of the vegetative body (mycelium) of the fungus *A. niger* allows the deacetylation of the chitin to prepare chitosan [16]. The extraction yield was 11.5% from 500 g of clean, dry mycelium. Although this yield is similar to those reported in the literature [17], it is low compared to the one obtained from crustaceans [18], since mycelium usually contain, in addition to chitin, different types of polysaccharides, proteins, and fatty acids [19].

Therefore, it is possible to generate CS with higher percentages if the extraction is performed from shells of crustaceans, since more than 60% of its content is chitin; however, there are some protein remnants left that may be allergenic, thus, it is preferred to use mycelium as a source of CS [20]. Moreover, shell extractions require pigment treatment with toxic solutions and enzymatic digestion that could increase the risk of contamination [18]. Mycelium, on the other hand, is a waste product of the industrial production of citric acid and is, therefore, a readily available raw material with lighter extraction conditions for the production of chitosan with lower environmental impact [18].

### 2.2. Deacetylation Degree and Characterization of CS by ^1^H-NMR

Extraction hydrolysis was performed with 40% NaOH, a concentration that generates a degree of chitin deacetylation greater than 50%, without breaking bonds and reducing molecular weight drastically. The deacetylation degree (DD) = 55.7%, was calculated by the potentiometric method (see Supporting Information, Figure S1) using Equation (1) [21].

NH_2_ (DD) = 16.1(y − x) × f/w (1)

where (y) and (x) represent the second and first volume of equivalence respectively, f is the base concentration, and w is the weight of CS used for titration. This method is not completely accurate, because it allows to manually quantify the free protons in solution, whose concentration varies depending on the ion strength [21]. NMR, on the other side, is a more accurate technique, because it is based on the absorption of all the protons of a molecule subjected to a magnetic field [22].

This method was used for the integration of the signals at δ = 4.74 and δ = 1.85 ppm, corresponding to the amine and methyl hydrogen of the acetamide group of CS, respectively [22], according to Equation (2):

DD = [100 − (IH(D)/(IH(A) − IH(D)))](100)(2)

where IH(D) corresponds to the proton integral in the amine of the deacetylated monomer and IH(A) corresponds to the proton in the methyl of the acetylated acetamide monomer. The calculation by this method gave a DD of 66.7%.

Finally, the ^1^H-NMR technique was used for the characterization of CS. In the spectrum (Figure S2), a signal appears at δ = 3.69 ppm for the anomeric proton of the deacetylated monomer and at δ = 3.48 ppm another signal appears for the anomeric proton of the acetylated monomer; in addition, two more signs are observed at δ = 4.74 and δ = 1.85 ppm, corresponding to the amine and methyl hydrogen of the acetamide group of CS, respectively [22].

### 2.3. Characterization of CS by FTIR Spectroscopy

The IR spectrum (Figure S3) shows the characteristic bands of the CS [22], for example, the band at 3360 cm^−1^ with a shoulder of 3273 cm^−1^ corresponding to the O-H stretching vibration of the glucosamine overlapped by the N-H vibration of the amine; in addition, at 1552 cm^−1^, the band due to the N-H stretching vibration of the primary amine of the deacetylated structure is observed. At 1639 cm^−1^ the band corresponding to C=O tension of the carbonyl of the acetylated monomer appears and finally, at 1076 cm^−1^, the band corresponding to the tension vibration of the C-O-C bond of the anomeric carbons [22].

### 2.4. Molecular Weight Determination of CS

To obtain the molecular weight, a capillary viscometer and a gel permeation chromatography (GPC) were performed on chitosan as it forms viscous solutions, allowing the measurement of the average viscous molecular weight (Mv) [23]. For this purpose, the drop times of 25 mL solutions were measured with a Ubbelohde viscometer, prepared from a standard solution (see supporting information). All the concentrations of the samples are shown in Table S1. The densities of the solutions and the constant β of the viscometer (3.31 × 10^−5^) were also calculated. From these data, the specific viscosity was calculated and plotted as a function of concentration (Figure S4).

The intrinsic viscosity is the intercept value of the specific viscosity curve and is symbolized by [η]. The Mark Houwink-Sakurada equation relates this viscosity to the constants *k* (1.81 × 10^−3^), and α (0.93), previously established for these conditions [24].

M_v_ = ([η]/K)^(1/α)^ (3)

The Mv calculated for the CS was 18,715 g/mol, as expected, lower than that contained in crustaceans [25].

The United States Pharmacopeial Convention (USP) guide for the chromatographic measurement of CS was used for the GPC analysis [26].

The peak corresponding to the CS appears at 22.3 min and with the calibration curve (Figure S5), the number average molecular weight (Mn) calculated was 6481.87 g/mol. This value is lower than the Mv, since Mn represents the total weight of all the molecules presented in the polymer sample divided by the total number of moles, while the Mv is related to a smaller quantity of moles: only those of low molecular weight [23].

### 2.5. Synthesis of the GO

An improved Hummers-Offemann method was used to obtain GO [27]. Typically, in the modified methods, solutions of H_2_SO_4_:HNO_3_ are used, and reactions are not to exothermic. For that reason, the reaction can take 1–2 weeks [27]. Marcano et al. found that using a 9:1 H_2_SO_4_:H_3_PO_4_ solution not only improves reaction performance, but also decreases reaction times to 6–12 h [27]. The use of the acid mixture allows the KMnO_4_ used for oxidation to react completely with the graphite in a exothermic reaction, and thus, increasing the performance and reaction speed. Any remaining of the oxidation is removed with H_2_O_2_ [27].

### 2.6. Characterization of the GO by FTIR Spectroscopy

**Figure 1:**
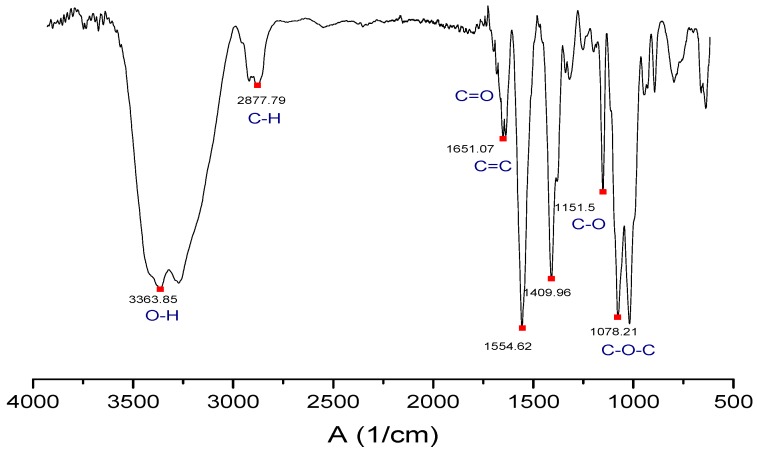
Fourier transform infrared spectroscopy (FTIR) spectrum of the graphene oxide (GO) synthesized in the study.

The IR spectrum (Figure 1) shows the characteristic bands of the GO [28]. The band at 3363 cm^−1^ corresponds to the O-H bond tension vibration, which is complemented by the C-OH band at 1151 cm^−1^ due to the hydroxyl groups of the GO. At 1651 cm^−1^ the tension band C=C characteristic of the double bonds in the polycyclic aromatic graphene ring appears. At 1078 cm^−1^ the C-O-C band characteristic of epoxy appears. The C=O band is displaced at a lower frequency by the interactions of the intramolecular hydrogen bond, which causes overlapping with the C=C band. Moreover, the band is weakened because the carbonyl groups are only around the sheet [28].

### 2.7. Characterization of the GO by Raman Spectroscopy

Raman spectrum (Figure S6) shows the two characteristic bands of the GO. The D = 1358 band shows the sp^2^-sp^3^ interactions between the GO carbons and the oxygen-rich groups, while G = 1598 band shows the sp^2^-sp^2^ interactions of the C=C links. The degree of oxidation of the GO was also measured by relating the intensity of both bands [29]. The degree of oxidation was found to be 80%, demonstrating that the procedure used to prepare GO was enough to obtain a high percentage of oxidation [29].

### 2.8. Characterization of GO by X-ray Diffraction (XRD)

**Figure 2:**
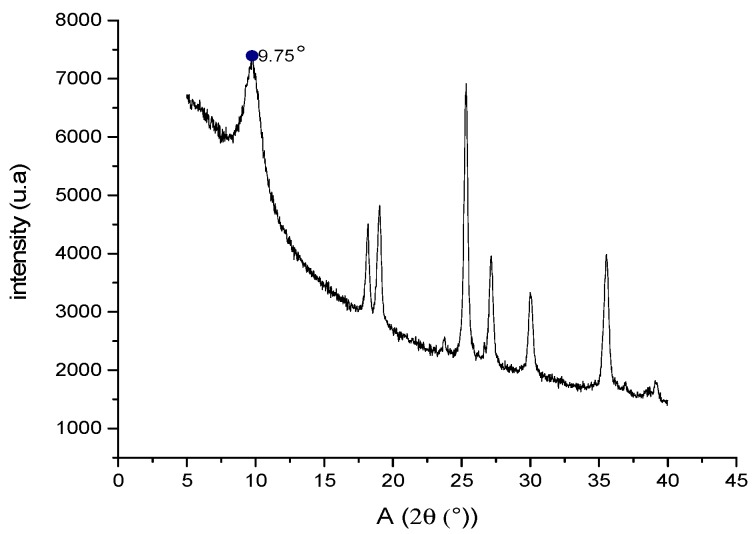
X-ray diffractogram of the GO synthesized in our study.

The GO diffractogram (Figure 2) shows the characteristic scattering peak at an angle of 9.75°. Usually, this peak appears at 10° [28] and represents the scattering caused by the -COOH groups that give the GO sheets an increase in size compared to graphite [28]. In the XRD spectrum, there are also other peaks between 20–40° which are characteristic of graphite oxide [28]; this indicates that there may be between two and ten overlapping GO layers when the GO is not dispersed in an aqueous solution. This factor loses relevance when preparing the scaffolds as the graphite oxide is dispersed, separating the sheets and reshaping the GO. This was verified by atomic force microscopy (AFM) studies, where measurements are made by dispersing the GO in solution.

### 2.9. Atomic Force Microscopy (AFM) Studies of GO

**Figure 3:**
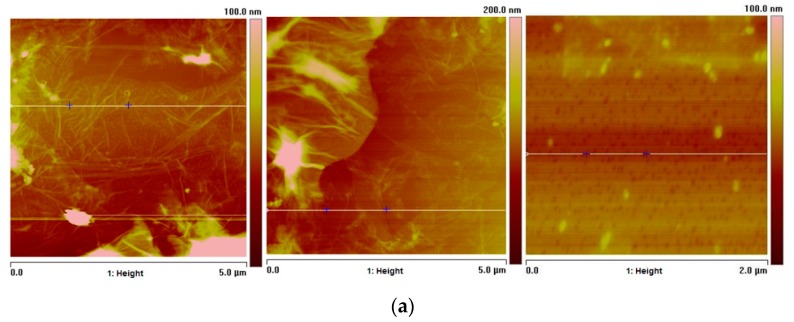

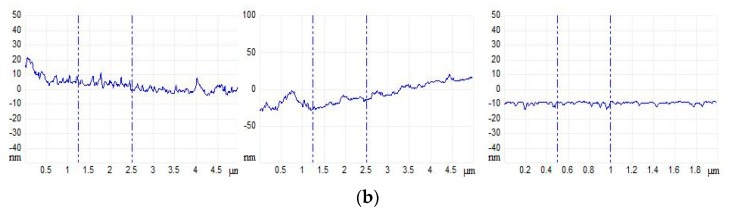
(**a**) Atomic force microscopy of sectioned GO plates (**b**) sectional thickness of each plate according to its height.

Figure 3, shows microscopic images of three different GO plates of the sample, with 5.0 µm and 2.0 µm area, dispersed in aqueous solution. The thickness profiles (Figure 3b) of one section of each sheet are shown. The thickness approaches to zero nm along the section since the thickness of a GO sheet must not exceed the thickness of a carbon atom [30]; however, there may be some agglomerations where thicknesses are of up to 20 nm. Independently, each film is individually dispersed in the solution.

### 2.10. Particle Size Study of GO by Dynamic Light Scattering

The particle size distribution of GO sheets was determined using Dynamic Light Scattering (DLS) (Figure S7). The average diameter of the sheets is 531.2 nm. Due to that, the size is low; the sheets do not overlap when dispersed in a solution allowing the construction of scaffolds without GO agglomerations.

### 2.11. Preparation of CS/GO Scaffolds by Freeze-drying Method

The scaffolds were prepared following a protocol similar to that of Mohandesa et al. [31]. Because the molecular weight of the CS obtained is low, the amount of CS used for scaffolding must be high [31]. For the same reason, the solution must be subjected to considerable agitation for the complete dispersion of the compounds.

The GO, on the other hand, is subjected to sonication to separate the overlapping layers (as shown in the XRD) to eliminate the agglomerations of the compound. This results in an efficient interaction between the CS and the GO where the roughness of the nanoscale surface of the GO, and the two-dimensional structure, establish an interaction that adheres the GO films to the CS chains, providing to the CS, strong mechanical properties that characterize the individual GO films [10].

Three formulations were used for the preparation of the scaffolds. One without GO and the other two with 0.5% and 1.0% GO to compare the morphology, thermal stability, biological potential of each scaffold, and to evaluate the effect of increasing the GO on the properties.

**Figure 4:**
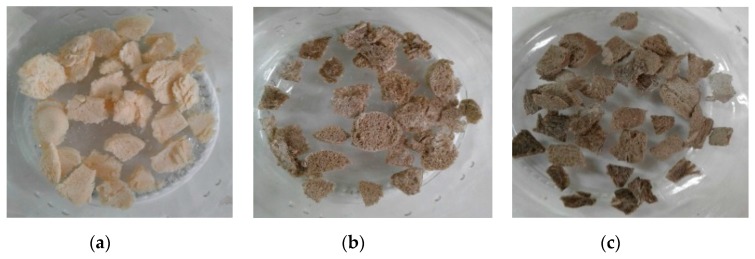
Pictures of the freeze-dried scaffolds of chitosan (CS) (**a**) without graphene oxide (GO), (**b**) with 0.5% of GO and (**c**) with 1.0% of GO.

After that, with the freeze-drying process, samples were frozen at low pressure, and the solvent was sublimated, producing a solid and stable structure (Figure 4) due to the strong interfacial adhesion between the well dispersed GO sheets and the polymeric matrix of CS [14]. The two-dimensional nanometric structure of GO finds it easy to align itself between the polymer chains. The hydrophilic characteristics of these films and CS give them a favorable compatibility with regard to the formation of hydrogen bonds evidenced in the FTIR [14], which results in homogeneous structures that are resistant to aqueous solvents, as shown by the hydrolytic degradation test, and whose porosity and roughness provide them with good scaffolding properties that stimulate cell growth [7].

### 2.12. Characterization of Scaffolds by FTIR Spectroscopy

**Figure 5:**
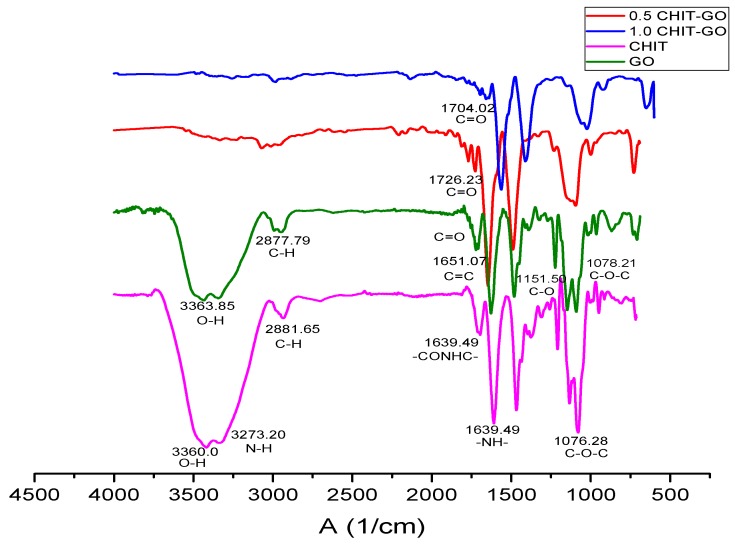
Fourier transform infrared spectroscopy (FTIR) spectrum of the chitosan/graphene oxide (CS/GO) scaffolds synthesized.

The IR spectrum of scaffolds (Figure 5) shows characteristic bands of both compounds. The signal of the band at a higher frequency of about 3360 cm^−1^, which for both compounds represents the O-H bond tension vibration, is weakened by the increase in the amount of GO.

This results from the interactions between the molecular vibrations of the hydroxyl of both compounds where the intensity of the overtones increases and the intensity of the fundamental band decreases [32]. This decreasing in the frequency of the band shows that there is a hydrogen bonding interaction between the hydroxyl of CS and GO [14]. The same occurs at 1070 cm^−1^ where the tension band of the C-O-C bond is located. Additionally, the epoxy groups interact at the same location with the OH groups of the CS [14]. This trend is not observed in the hydroxyl band, because the C-O-C band is also part of the glycosidic bond and these oxygenate groups are not susceptible to interaction by this type of bond [14].

In turn, between 1704 cm^−1^ and 1726 cm^−1^ the band corresponding to the C=O appears at a lower frequency due to the intramolecular hydrogen bonds of the GO, which was previously weakened [28]. However, for the scaffolds, carboxyl oxygen is intermolecularly bonded with the oxygenated molecules of the CS and therefore its frequency increases and no longer overlaps with the C=C band, leaving the carboxyls of the GO evident [14].

The other bands that are not part of the hydrogen interactions such as the C-C bond tension band and the -CONHC- of the substituted N- amide remains intact [14].

### 2.13. Scanning Electron Microscopy (SEM) of Scaffolds

**Figure 6:**
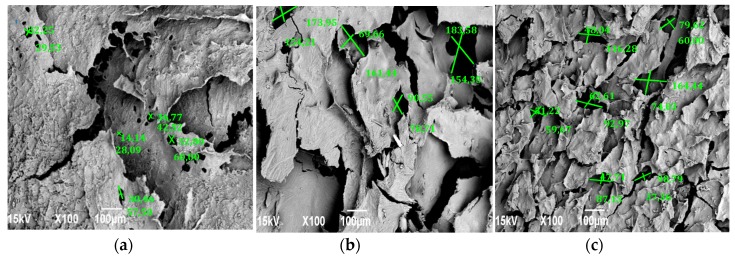
Scanning electron microscopy of chitosan (CS) scaffolds (**a**) without graphene oxide (GO), (**b**) with 0.5% GO and (**c**) with 1.0% GO.

Figure 6 shows microscopic images of the scaffolds prepared with the three formulations, respectively.

Each composition shows porosity and interconnection, however, the morphology varies as the amount of GO is increased. Figure 6a which corresponds to the CS in the absence of GO, less porosity and roughness is perceived as compared to the other samples. Additionally, most of these pores do not exceed 50 µm, a size necessary for cell proliferation [33]. For this reason, cells are less likely to clump together in this structure; Figure 4b,c, containing 0.5% and 1.0% GO, have more pores and flakes. The formulation containing 1.0% of GO shows more homogeneity with the porosity with an average size of 78.38 µm, calculated using the pore diameter Equation (4) [34],

d = ((l)(h))^(1/2)^ (4)

where l and h are the maximum and minimum pore size, exceeding the threshold for cell growth. This homogeneous structure, which could promote optimal cell proliferation, is the result of the interaction of CS and GO confirmed by the FTIR and SEM, whose hydrophilic interactions [14] and three-dimensional shape provides stability as confirmed by other authors [7,34]. This was verified with the thermogravimetric analysis and the degradability test.

### 2.14. Thermogravimetric Analysis of Scaffolds

Thermogravimetric analyses (TGA) of the scaffolds are shown in Figure S8. The thermogram of the CS scaffold without GO (a) shows two mass losses during the temperature rise, as normally occurs in the TGA of the non-lyophilized polymer [35]. The first loss (26.5%) occurs between 71.1 °C and 128.6 °C and corresponds to the remaining mass of water in the CS that is not dry, even after freeze-drying. This loss is also noticeable in scaffolds with 0.5% GO Figure S8b and 1.0% GO Figure S8c; however, in (c), less than half of the water mass degraded in (a) is lost, due to the fact that as the temperature increases, these nanocomposites tend to behave like hydrogels that retained water molecules in their matrices [36]. The hydrogen bonds between the two compounds were weakened, allowing CS chains to stretch freely, which increased the possibility of interacting with other GO sheets, generating greater intercrossing and a water-related three-dimensional network. Thus, it was not easily eliminated [36].

The second loss (33.8%) of the mass of the scaffold without GO (a) with a maximum degradation of 253 °C corresponds to the degradation of the CS chains [35]. In comparison, for the scaffolds containing GO, the decomposition temperature was 270.5 °C and 273.9 °C for (b), and (c), respectively. This shows that there was a strong interaction, due to the hydrogen bonds between the chains of CS and GO [10], which gives thermal stability of a polymer with a higher molecular weight [36].

For scaffolds with GO content, a third degradation temperature with losses of 19.3% and 17.6% at 485.6 °C and 481.8 °C for (b) and (c), respectively, were observed. This is attributed to the removal of the stable oxygenated functional groups of GO [29] and the removal of aromatic remains [26]. Thus, this degradation did not occur in scaffolds without GO content [29]. It is important to remark that most of the weight loss occurred at temperatures above human body temperature, which indicates that all these structures will be very stable under body conditions.

### 2.15. Degradability Test of Scaffolds on Physiological Serum

Figure S9 shows the scaffolds added in aqueous medium sterilized at 0.90% *w/v* NaCl. The scaffold degradability (solubility) in the serological medium was evaluated qualitatively in this environment. Usually, increased stability with the introduction of GO, an essential fact for cell proliferation, is expected in in vitro biological tests.

The scaffold of CS without GO (a) was completely dissolved in the physiological medium within five minutes, while the scaffolds whose compositions contain GO did not dissolve in the medium, as a result of the improved mechanical properties that GO provides [7]. The introduction of GO increased stability with the increase of GO percentage (b and c).

### 2.16. Biological Tests

After remaining implanted for 30 days, the samples were recovered and studied macroscopically and by using optical microscopes.

**Figure 7:**
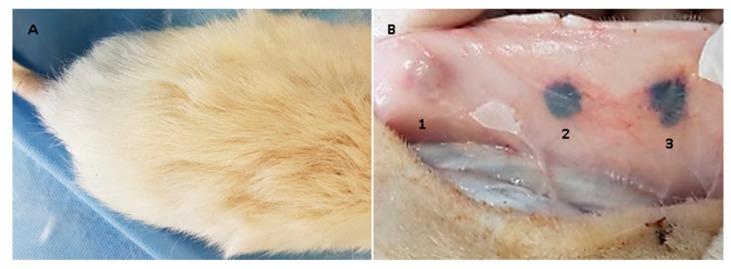
Image (**A**) shows the dorsal area where the surgical preparation was made; image (**B**) shows the internal surface of the skin, the three samples encapsulated by a scar tissue are appreciated. 1: chitosan/graphene oxide (CS/GO) 0%; 2: CS/GO 0.5% and 3: CS/GO 1%.

When the dissection was performed, it was observed that the skin had generally healed with hair formation and, inside it, the material remained encapsulated, without the presence of granulation tissue or purulent exudate (Figure 7).

**Figure 8:**
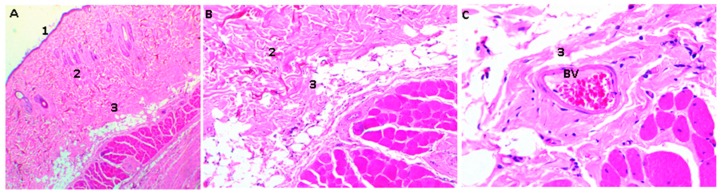
Rat skin sample implanted with collagen film. 1: Epidermis, 2: Dermis, 3: Subcutaneous cellular tissue. Three corresponds to the implantation area, BV: Blood vessel. Hematoxylin-Eosin Technique. Image (**A**) is at 4× magnification, Image (**B**) at 10×, and Image (**C**) at 40×.

The samples were processed for histological analysis by Hematoxylin and Eosin (HE) and Masso trichromacy (MT) techniques, where a porcine collagen film of 5 mm diameter by one mm in thickness was used as control material [37]. The studies with HE showed the presence of a normal histological architecture in the implantation zone (subcutaneous cellular tissue); additionally, there was no control material present (Figure 8).

Unlike the samples containing the control material, CS and CS/GO scaffolds were found in different resorption states in all the experimental samples. It is evident that the higher the GO content, the less evidence of degradation is observed, as expected for tissue regeneration applications [37].

**Figure 9:**
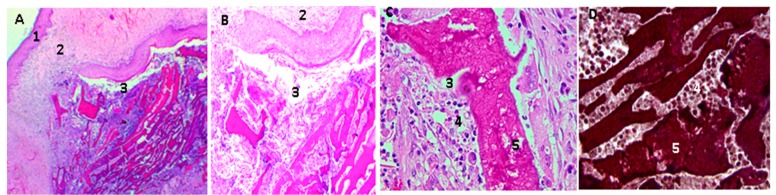
Chitosan/graphene oxide (CS/GO) 0% films implanted in rat skin; in the pictures (**A**,**B**), movies are seen at 4× and 10×, respectively, using the Hematoxylin-Eosin Technique; in the images (**C**,**D**), at 40×, the films in the process of and surrounded by a mixed inflammatory infiltrate are observed, the D image is realized using Masson’s Trichromacy technique. 1: epidermis, 2: Dermis. 3: subcutaneous cellular tissue (implantation zone), 4. Mixed inflammatory infiltrates, 5. Scaffolds of CS/GO 0%.

Figure 9 shows the histological analysis of a CS/GO 0% scaffold. CS scaffolds were observed in an evident degradation/resorption process; there was also the presence of mixed inflammatory infiltrate, responsible for the resorption.

**Figure 10:**
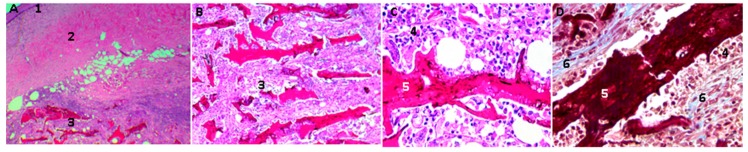
Scaffolds of chitosan/graphene oxide (CS/GO) 0.5% implanted in rat skin; in the images, (**A**,**B**) scaffolds are appraised to 4× and 10×, respectively, using the Hematoxylin-Eosin Technique. Images (**C**,**D**), at 40×, are scaffolds in the process of degradation and are surrounded by a mixed inflammatory infiltrate. Image (**D**) is made using Masson’s trichromacy technique; in this image we can see a fibrous tissue capsule surrounding the CS/GO scaffolds. 1: epidermis, 2: Dermis. 3: subcutaneous cellular tissue (implantation zone), 4. Mixed inflammatory infiltrates, 5. Scaffolding CS/GO 0.5%, 6: Fibrous capsule. Technique. Image (**A**) is at 4× magnification, Image B at 10×. Image (**C**) at 40×.

In Figure 10, scaffolds of CS/GO 0.5% are shown after 30 days of implantation: there was less evidence of resorption/degradation than in the case of films of only CS. Additionally, there was abundant inflammatory infiltrate.

**Figure 11:**
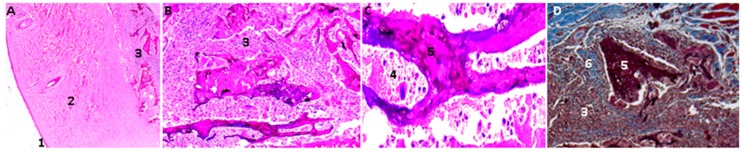
Scaffolds of CS/GO 1% implanted in rat skin. In the images (**A**,**B**), the scaffolds are appreciated at 4× and 10×, respectively, using the Hematoxylin-Eosin Technique. In the image (**C**) at 40×, the film is observed in the process of resorption and surrounded by a mixed inflammatory infiltrate. The image (**D**) at 10× is made using Masson’s trichromacy technique. In this image, a fibrous tissue capsule is seen surrounding the scaffold of CS/GO. 1: Epidermis, 2: Dermis. 3: subcutaneous cellular tissue (implantation zone), 4. Mixed inflammatory infiltrates, 5. CS/GO 1%, 6: Fibrous capsule.

The CS/GO, 1% scaffolds present less evidence of resorption/degradation as shown in Figure 11. There is also an abundant inflammatory infiltrate and a fibrous capsule surrounding the particles.

The macroscopic findings (normal scarring and formation of new skin hair) and the encapsulation of the embedded materials without signs of granulation tissue, or the presence of purulent exudate, but also with the recovery of standard tissue architecture, are evidence of the initial biocompatibility of the material [38].

The presence of a fibrous capsule surrounding the material in the process of resorption, as well as the inflammatory infiltrate observed in all cases, indicate a process of cell-mediated resorption, which is in the parameters of normality of a standard resolution process for these types of materials, mediated by a reaction response to a foreign body [38].

Although there is no greater availability of research where histological studies of graphene oxide implanted in biomodels were carried out, it is possible to find works with in vivo characterizations, similar to the one used in this research, for different materials considered biocompatible, such as calcium phosphate, hydroxyapatite, and chitosan.

Cruz et al. and Gonzales et al. determined the biocompatibility of a tricalcium phosphate and chitosan bio-composite implanted in wistar rats’ subdermal tissue for 20 days. They found an inflammatory infiltrate and a connective tissue surrounding the material [39]; similarly, Figueiredo et al. compared, in an eight-day study, the intramuscular inflammatory response in Wistar rats, from a xenograft (porcine origin) and an alloplastic (hydroxyapatite), finding a moderate inflammatory response and forming a fibrous capsule [40]. Broon et al. inserted a bio-ceramic material in wistar rats subdermal tissue, observing a mild to moderate inflammatory response with the presence of collagen fibers at 20 days [41].

Additionally, Balanta, Zuluaga, and Valencia found that after 21 days of implantation of chitosan films in wistar rats, subdermal tissue remained without reabsorbing surrounded by a fibrous tissue capsule [42]. Moura et al. implanted chitosan hydrogels, finding a chronic inflammatory response at 30 days with the presence of mixed inflammatory infiltrate and a fibrous capsule surrounding the material [43].

The results presented some materials as biocompatible (calcium phosphate, hydroxyapatite, and chitosan), which indicates that the most common inflammatory response for biomaterials implanted in subdermal tissue of rats is the chronic inflammatory reaction, characterized by an inflammatory infiltrate between mild and moderate, with presence of a fibrous capsule surrounding the material.

The toxicity of graphene oxide has been studied mainly in cell cultures, finding that some cell lines are more sensitive than others to the effect of the nanoparticle (intracellular localization and toxicity of graphene oxide and reduced graphene oxide nanoplatelets to mussel hemocytes in vitro) [44]. According to Lalwani et al., the cytotoxicity of the GO is influenced by the size of the particular, dose, distribution, obtaining technique, etc. [45]. In the case of this investigation, large particles were used, in a range between 50 and 1000 μm. This could have influenced the response to the foreign body that was present and in the encapsulation of the particles by a fibrous capsule while the process of cell-mediated resorption was carried out [44], as well as the functionalization with the CS on what improved the biocompatibility of the compound.

Thangave et al. used a dorsal skin design of murine models, and found eight days of skin scarring with collagen formation stimulus when using scaffolds of reduced graphene oxide ((RGo)-Isabgol in normal and diabetic rats) [37]. In this investigation, with 30-day results, the normal healing process was observed with the formation of a collagen capsule surrounding the particles and the presence of blood vessels.

## 3. Experimental

### 3.1. Materials

The mycelium of the *Aspergillus niger* fungus was obtained from Sucroal (Cali, Colombia). All other reagents were used as obtained from Sigma-Aldrich (Palo Alto, CA, USA) unless otherwise stated.

### 3.2. Synthesis

#### 3.2.1. Extraction and Purification of CS

Of the mycelium of the *A. niger*, 500 g was placed in three beakers, and 2 L of deionized water was added. Each solution was mixed using a mechanical mixer, Heidolph RZR 2020 (Heidolph Instruments, Schwabach, Germany), to wash the mycelium. After that, it was filtered off. The mycelium was harvested, and the washing process was repeated six times to remove impurities that were not part of the mycelium.

The mycelium resultant was dispersed in two trays and allowed to dry at 90 °C for 48 h in an oven. Then, it was added to a 5 L nozzle balloon, and after that, it was added to a 40% solution of NaOH in a 5:1 ratio relative to dry mycelium. This solution was mechanically stirred at 1300 rpm for 5 h at 120 °C and then removed and filtered after completion of the reaction.

The brownish filtrate corresponding to hydrolyzed mycelium was collected and washed according to the washing protocol described above until it reached a pH close to 8.

The mycelium obtained was introduced in a beaker and 3 L of water were added. The solution was stirred at 1300 rpm, and glacial acetic acid was added dropwise until a pH of 3.8 was reached to dissolve the CS. This solution was filtered again, and the process was repeated until a pH of 9.8 was reached to precipitate the CS.

The CS was filtered and dried for 24 h at 60 °C. To purify it, it was solubilized again with acetic acid and precipitated with NaOH, then filtered again (11.5% yield) [13].

#### 3.2.2. GO Synthesis

In a 500 mL Erlenmeyer, placed in a refrigerator with ice, three grams of graphite, nine grams of KMnO_4_ and 100 mL of an H_2_SO_4_:H_3_PO_4_ solution, were added slowly with constant magnetic agitation and mixed together. After that, this solution was sonicated for 3 h in a sonicator, Branson 2800, (Branson Ultrasonics Corporation, North Olmsted, OH, USA) with cold water reflux to prevent the temperature from exceeding 25 °C. Then, another nine grams of KMnO_4_ was added and sonication was continued for three more hours without allowing the temperature to exceed 40 °C. A 0.013 mM solution of H_2_O_2_ was added until effervescence was observed, after which it was washed with H_2_O-miliQ in a centrifuge until a neutral pH was reached. A final washing with CH_3_OH ≥ 99% was carried out and it was then dried in a vacuum furnace, (Precision Scientific CO, Chicago, IL, USA) at 60 °C for 2 h to obtain 5 g of GO [27].

#### 3.3.3. CS/GO Scaffold Preparation

The CS was pulverized and sieved to achieve uniform particle size with a D-500 Success Technic disperser (Success Technic Industries, Selangor, Malaysia). GO, on the other hand, was dispersed in water and sonicated for 30 min to cause exfoliation and separation of the slices. Both compounds were dispersed and agitated for 1 h at 10,000 rpm in dispersing equipment to make three solutions: 19.5% CS/GO and 2% CH_3_COOH by adding 0, 0.5 g, and 1 g GO respectively [31].

For each formulation, two falcon tubes were completed with 30 mL of the solution, which were then frozen in a freezer at −9.8 °C for 24 h.

The six tubes were then placed in a freeze-dryer Labconco Freezone 4.5 (Labconco Corporation, Kansas City, MO, USA) at a pressure of 12 Pa and −52°C for 48 h to sublimate the CH_3_COOH solvent and generate the scaffolds completely. Finally, these were cut with a scalpel to 1 mm thick and approximately 5 mm in diameter for characterization, for performing the degradability test in aqueous serum at 0.90% *w/v* NaCl, and for the insertions in bone and rat skin.

### 3.3. Characterization

^1^H-NMR measurements were performed on a 400 MHz NMR Bruker Ultra Shield (Bruker Corporation, Billerica, MA, USA) using DMSO-d6 as a solvent. FTIR measurements were performed on an infrared equipment, Thermo brand model NIcolet 6700 (ThermoFisher Scientific, Waltham, MA, USA), using KBr tablets. GPC measurements were performed using a gel permeation chromatograph Agilent 1200 (Agilent Technologies, Santa Clara, CA, USA) with 2 intercrossed polymer columns Shodex ohpak (Showa Denko, Tokyo, Japan) as the stationary phase and NaNO_3_ 0.15 M /HCOOH 0.5 M as the mobile phase, using pullulan standards for the calibration curve (Figure S4), and a refractive index detector. Raman determination was performed on a Confocal Raman Microscope (Renishaw InVia Reflex, Wotton-under-Edge, UK). XRD measurements were run on an diffractometer X’pert PRO (Malvern PANalytical, Jarman Way, Royston, UK) Radiation: k(alpha1) 1.540598 and k(alpha2) 1.544426, from a copper anode with a 45 kV electron accelerator voltage and current to generate 40 mA electrons, with 0.25° and 0.125° incident beam optical grid, 75 mm diffracted beam grid, 0 mm soler grid.04 rad and a detector PIXel (Malvern PANalytical, Jarman Way, Royston, UK) in Scanning mode with an active length (°) of 2.5108, step size 0.0197°, range of 2θ from 4°–90°, and time per step 304.390 s. The AFM analysis was performed in a multimode AFM, Vecco Instruments (Santa Barbara, CA, USA) equipped with a nanoscope Iva control system version 6.14r1 (Vecco Instruments, Santa Barbara, CA, USA; particle size measurements. DLS was measured on a particle size determiner, Zetasizer Nano Z (Malvern Panalytical, Jarman Way, Royston, UK) Scanning electron microscopy (SEM) was performed on a scanning electron microscope (JEOL JSM-6490LA, Musashino, Tokyo, Japan) and each sample was coated with a copper bath. The Thermogravimetric analysis was performed on a TGA-2050 thermogravimetric analyzer (TA instrument, New castle, DE, USA) adjusted in a working temperature range between 25–400 °C.

### 3.4. In Vivo Compatibility Tests

Following the ISO 10993 standard recommendations, subdermal implantation tests were performed with three samples of each formulation on the dorsal surface of three adult Wistar rats. For the implantation, a surgical preparation was made on the skin, 10 mm long by 15 mm deep and 0.02 g of each formulation were deposited. Finally, the tissues were closed with silk suture 4-0. We then waited for 30 days, to observe the tissue reaction to the implanted materials. This research was approved by the animal ethics review committee of Universidad del Valle in Cali, Colombia, according to the endorsement of the ethics committee CEAS 001-016.

## 5. Conclusions

The degree of deacetylation of 55.7% and 66.7%, calculated by potentiometric and ^1^H-NMR method showed that the chitin contained in the mycelium of the *A. niger* reacted, producing chitosan with a yield of 11.5% based on the initial dry weight.

The graphene oxide was synthesized by an improved Hummers-Offemann method. The improvements get an increase in the weight of the GO, higher than the weight of the starting material. The FTIR and the Raman spectra demonstrated that the oxidation was completed. The AFM and XRD images showed that graphene oxide was obtained after the freeze-drying method.

Scaffolds were successfully elaborated using the freeze-drying method. Their FTIR spectrum showed a decrease in the intensity and the frequency of the characteristic bands of the oxygen groups of both compounds. This indicates that interaction by hydrogen bonds between both molecules occurs as the amount of GO increases.

The TGA and serum degradation test corroborated this interaction by showing that scaffolds with larger GO compositions tend to degrade less; this also showed that absorption in dermal tissues is greater once the scaffolds are implanted. SEM images showed that the morphology, interconnection, and uniform porosity of the scaffolds with higher GO content allow them to have the potential to generate cell growth of osteoblasts so that they can be used for future biocompatibility tests and insertions in living organisms.

The three formulations behaved as biocompatible in the 30 days that the materials were implanted. Histological analysis showed a decrease in resorption and degradation related to an increase of the GO in each scaffold. Macroscopically, the material presented compatibility, which is evidenced in the formation of new hair, in wound healing, and the recovery of tissue architecture. Moreover, there was no presence of granulation with tissue damage or the presence of pus, which evidenced the initial biocompatibility of the material.

The implanted materials were surrounded by a fibrous capsule, in a typical reaction to a foreign body and resorption of the material mediated by inflammatory cells.

All these results suggest the potential that CS-GO scaffolds obtained have in tissue engineering for cell regeneration starting from a CS obtained from the *A. niger* mycelium, derived from the residue of a company producing citric acid.

## Supplementary Materials

The following are available online. Table S1: Concentrations and drop times of solutions taken in an ubbelohde viscometer, Figure S1: Potentiometric titration of the CS, Figure S2: 1H-NMR of the CS, Figure S3: FTIR of the CS, Figure S4: Viscosity curve. Specific viscosity vs. concentration, Figure S5: Calibration curve of GPC with pululan standards, Figure S6: Raman of the GO, Figure S7: DLS of the GO, Figure S8: The thermogravimetric curve of CS scaffolds (a) without GO, (b) with 0.5% GO and (c) with 1.0% GO, Figure S9: Degradability test of CS scaffolds in physiological serum (a) without GO, (b) with 0.5% GO and (c) with 1.0% GO added.
