# Supplementary material for: Synthesis and Application of Scaffolds of Chitosan-Graphene Oxide by the Freeze-Drying Method for Tissue Regeneration
Source: Molecules. 2018 Oct 16;23(10):2651. doi: 10.3390/molecules23102651 (PMC6222393; doi:10.3390/molecules23102651)
Supplement: Supplementary file 1 [file molecules-23-02651-s001.pdf]

molecules

Article

Synthesis and application of scaffolds of Chitosan-Graphene oxide by the Freeze-drying method for tissue regeneration

Cesar Valencia [1], Carlos H. Valencia[2], Fabio Zuluaga[1], Mayra E. Valencia[3], José H. Mina[3], and Carlos David Grande-Tovar[4*]

[1] Laboratorio SIMERQO polímeros, Departamento de Química, Universidad del Valle, Calle 13 # 100-00, Cali, Colombia
[2] Escuela de Odontología, Grupo biomateriales dentales, Universidad del Valle, Calle 13 # 100-00, Cali, Colombia
[3] Grupo de Materiales Compuestos, Escuela de Ingeniería de Materiales, Universidad del Valle, Calle 13 # 100-00, Cali, Colombia
[4] Grupo de Investigación de Fotoquímica y Fotobiología, Universidad del Atlántico, Carrera 30 Número 8-49 Puerto Colombia, Colombia
* Corresponding author: carlosgrande@mail.uniatlantico.edu.co

Received: date; Accepted: date; Published: date

1. Experimental

1.1. Deacetylation degree and characterization of CS by potentiometric method

For the DD calculation, a potentiometric titration of CS with a WTW pHmeter pH 3310 was performed, a NaOH solution of 0.101 M previously standardized with $C_8H_5KO_4$ as titrating agent was used and a solution of 0.253 g of CS in 20 ml HCl 0.3M.

1.2. Capillary viscosimetry for the calculation of the Mv.

A standard solution of 100 mL of CH_3COOH 0.1 M, NaCl 0.2 M and 0.02 g/mL of CS was prepared for the calculation of the Mv of CS. From this 5 solutions of 25 mL were prepared with the concentrations shown in figure 3 and the density was measured with a pycnometer of 1 mL and the fall time with a viscometer of ubbelohde viscometer with constant β of $3.31x10^{-5}$ that is calculated with the time of fall of the water.

1. Supporting tables

Table S1. Concentrations and drop times of solutions taken in an ubbelohde viscometer.

Concentration (mL/g)	Time (s)	ρ (g/mL)
Solvent	277	1.0320
0.0008	279	1.0324
0.0010	282	1.0327
0.0013	283	1.0332
0.0015	284	1.0348
0,0018	285	1.0360
0.0020	286	1.0368

2. Supporting images

molecules

MDPI

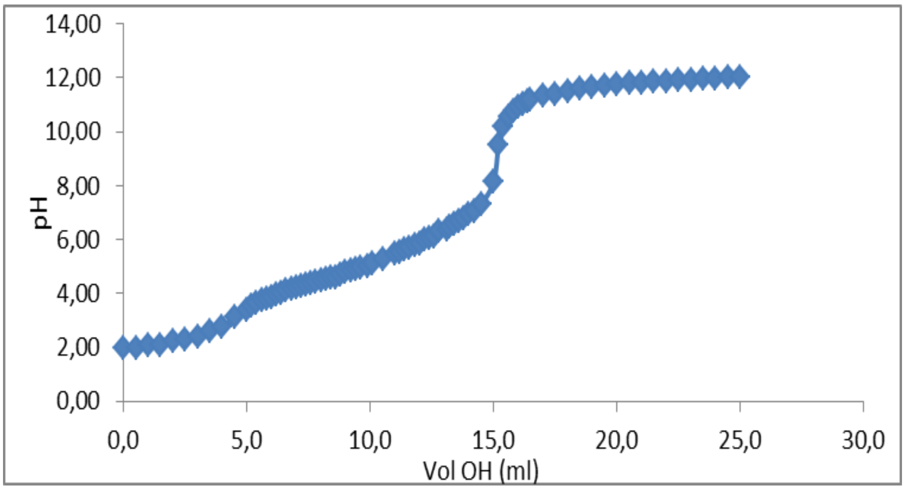

Figure S1. Potentiometric titration of the CS.

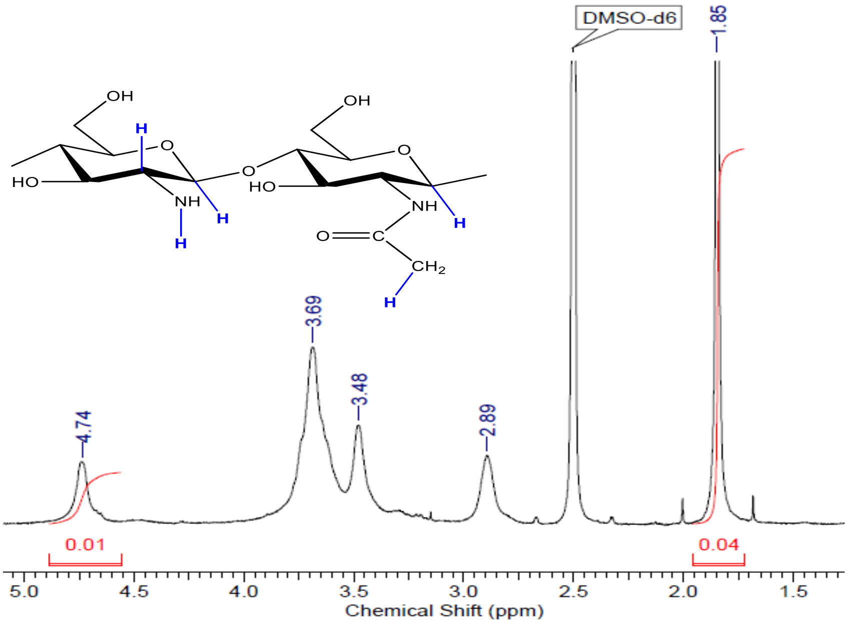

Figure S2. ¹H-NMR of the CS.

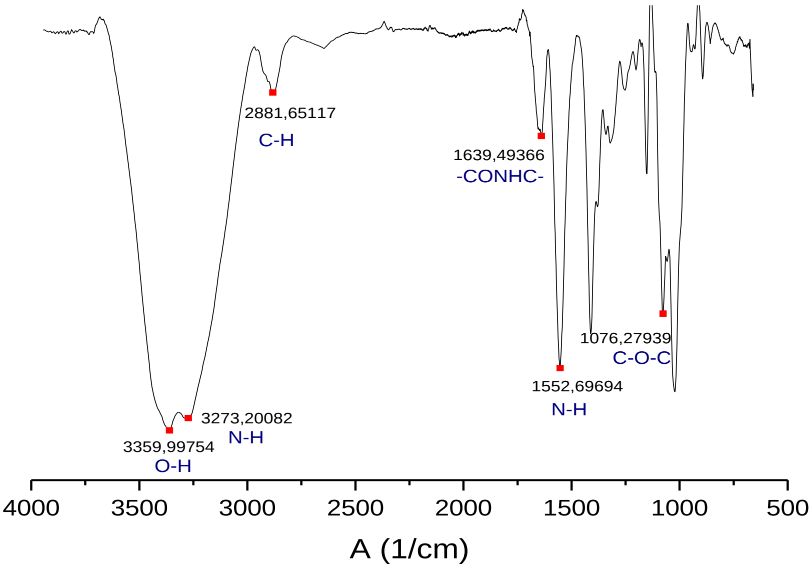

Figure S3. FTIR of the CS.

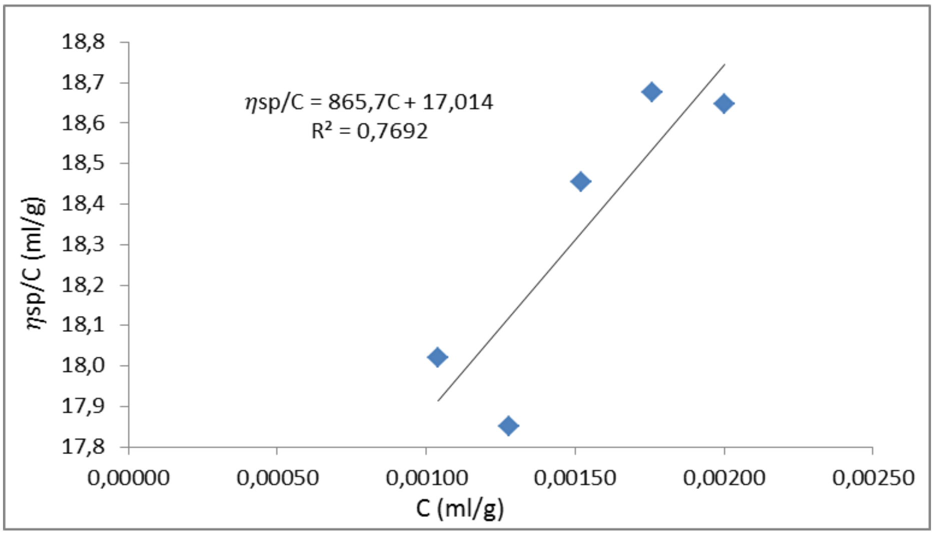

Figure S4. Viscosity curve. Specific viscosity vs concentration.

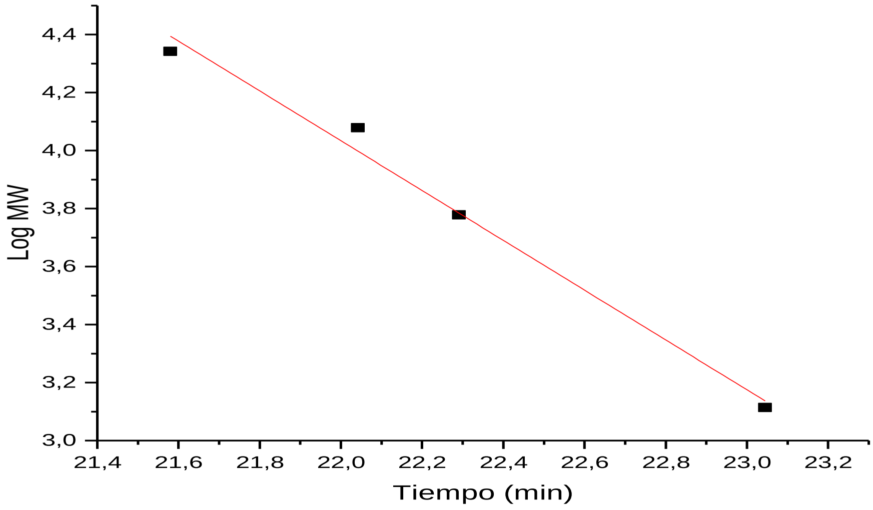

Figure S5. Calibration curve of GPC with pululan standards.

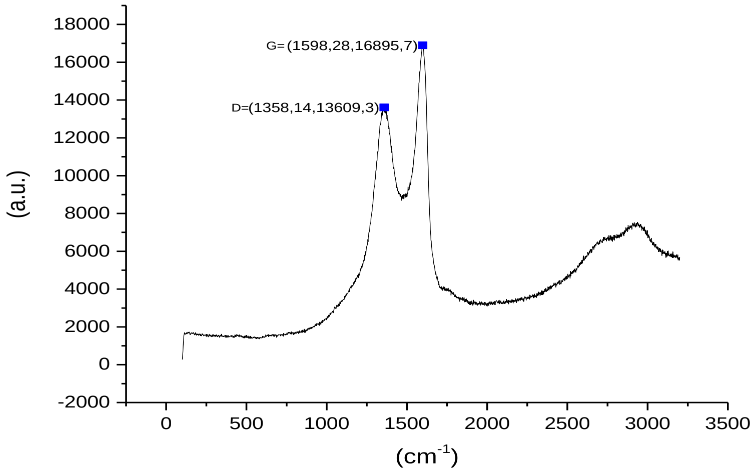

Figure S6. Raman spectrum of the GO

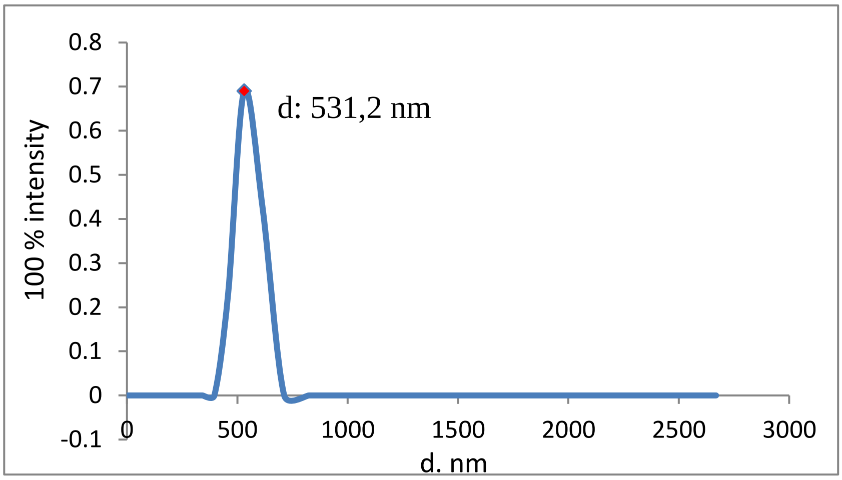

Figure S7. DLS of the GO

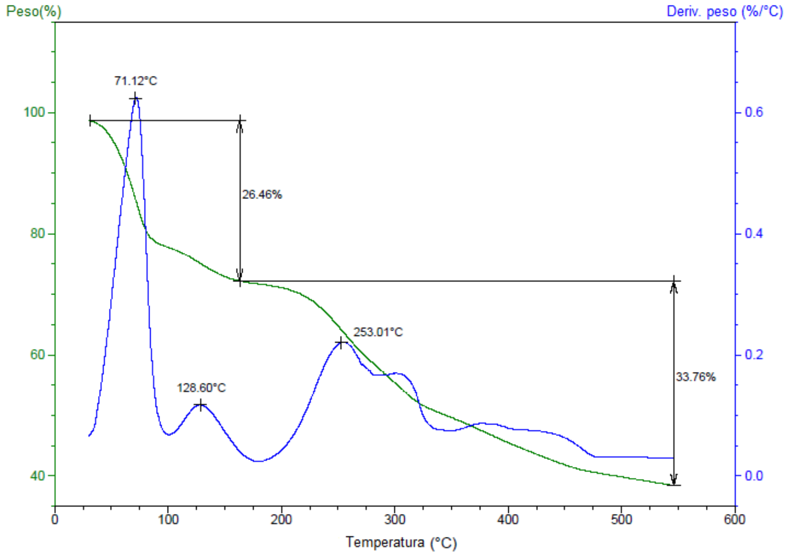

(**a**)

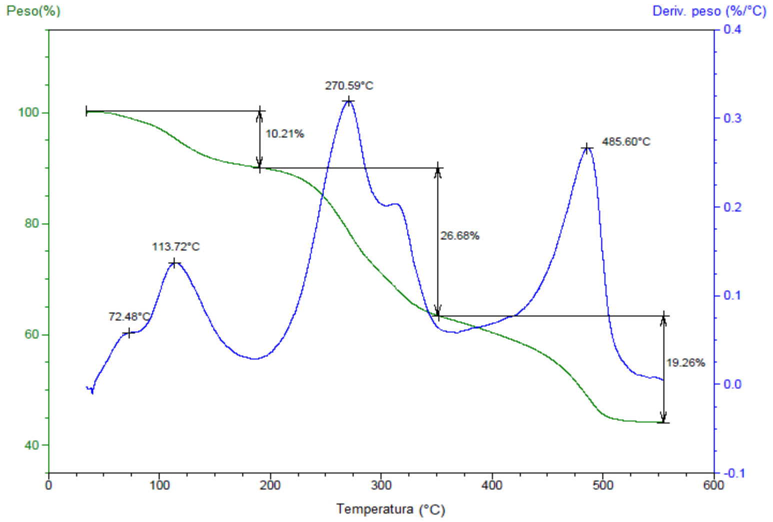

(**b**)

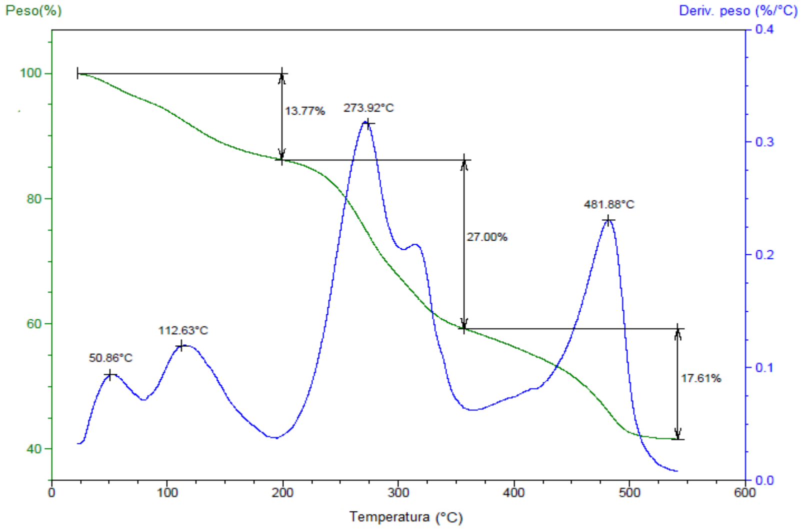

(**c**)

Figure S8. Termogravimetric curve of CS scaffolds (a) without GO, (b) with 0.5% GO and (c) with 1.0% GO.

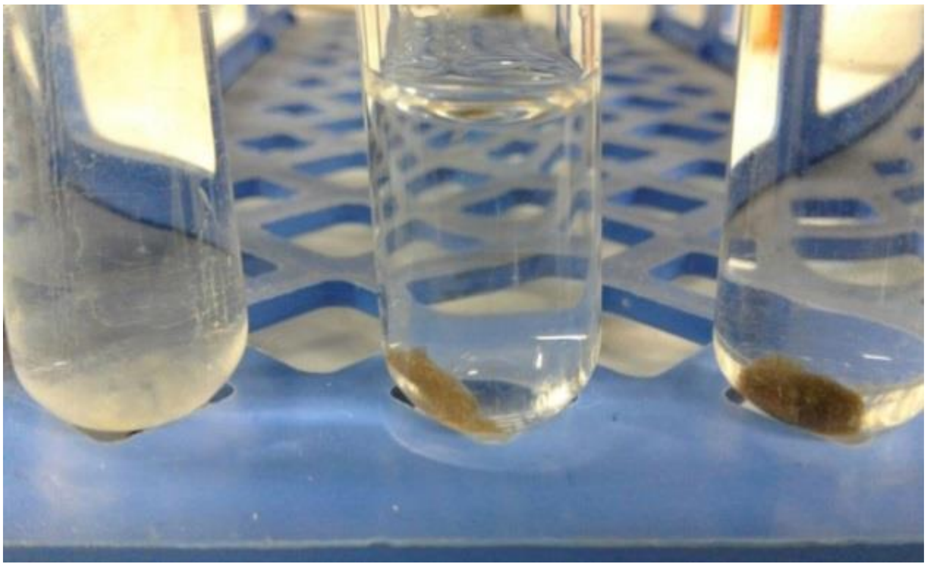

(**a**) (**b**) (**c**)

Figure S9. Degradability test of CS scaffolds in physiological serum (a) without GO, (b) with 0.5% GO and (c) with 1.0% GO added.
